# Alcohol-specific transcriptional dynamics of memory reconsolidation and relapse

**DOI:** 10.1038/s41398-023-02352-2

**Published:** 2023-02-15

**Authors:** Koral Goltseker, Patricia Garay, Katherine Bonefas, Shigeki Iwase, Segev Barak

**Affiliations:** 1 School of Psychological Sciences, Tel Aviv University, Tel Aviv, 69978 Israel; 2 Zuckerman Mind Brain Behavior Institute, Columbia University, New York, NY 10027 USA; 3 The University of Michigan Neuroscience Graduate Program, Ann Arbor, MI USA; 4 Human Genetics Department, The University of Michigan Medical School, University of Michigan, Ann Arbor, MI 48108 USA; 5 Sagol School of Neuroscience, Tel Aviv University, Tel Aviv, 69978 Israel

**Keywords:** Molecular neuroscience, Addiction

## Abstract

Relapse, a critical issue in alcohol addiction, can be attenuated by disruption of alcohol-associated memories. Memories are thought to temporarily destabilize upon retrieval during the reconsolidation process. Here, we provide evidence for unique transcriptional dynamics underpinning alcohol memory reconsolidation. Using a mouse place-conditioning procedure, we show that alcohol-memory retrieval increases the mRNA expression of immediate-early genes in the dorsal hippocampus and medial prefrontal cortex, and that alcohol seeking is abolished by post-retrieval non-specific inhibition of gene transcription, or by downregulating ARC expression using antisense-oligodeoxynucleotides. However, since retrieval of memories for a natural reward (sucrose) also increased the same immediate-early gene expression, we explored for alcohol-specific transcriptional changes using RNA-sequencing. We revealed a unique transcriptional fingerprint activated by alcohol memories, as the expression of this set of plasticity-related genes was not altered by sucrose-memory retrieval. Our results suggest that alcohol memories may activate two parallel transcription programs: one is involved in memory reconsolidation in general, and another is specifically activated during alcohol-memory processing.

## Introduction

Alcohol use disorder (AUD) is a detrimental neuropsychiatric disorder with severe medical, social, and economic burdens [1], yet available pharmacotherapy is limited [2]. Nearly 70% of patients relapse within the first year of abstinence [3], marking relapse as a major clinical challenge. Relapse is often triggered by craving for alcohol, evoked by environments and cues previously associated with alcohol [4]. Therefore, the disruption of memories that evoke alcohol-related behaviors is expected to reduce or even prevent cue-induced relapse [5, 6].

It is increasingly accepted that well-consolidated memories can be reactivated upon retrieval. Retrieved memories undergo temporary destabilization and subsequent re-stabilization, a process termed reconsolidation [7–11]. Thus, memory reactivation initiates a temporary “reconsolidation window”, lasting a few hours, during which a memory is labile for certain manipulations [7, 8, 11, 12]. Indeed, interference with the reconsolidation of drug memories was shown to attenuate their subsequent expression and cue-induced relapse, thus providing a potential strategy for relapse prevention [13, 14].

Although the exact mechanisms underpinning the processing of reactivated drug memories have yet to be characterized, reconsolidation of drug and alcohol memories was generally shown to be interrupted by the inhibition of NMDA [15–17] or beta-adrenergic receptors[17, 18]; or by preventing protein synthesis [5, 10, 15]. According to recent fear and drug memory studies, memory reconsolidation requires not only protein synthesis but also gene transcription [19]. Moreover, the transcription of certain immediate early genes (IEGs), including *Arc*, encoding activity-regulated cytoskeleton-associated protein and the transcription factor-encoding *Egr1* (*Zif268*), was implicated in the reconsolidation of various types of memory [19–22], implying that similar dynamics might control the reconsolidation of alcohol memories. Similarly, we previously showed that inhibition of mechanistic target of rapamycin complex 1 (mTORC1), which controls the synthesis of a subset of dendritic proteins [23], disrupted the reconsolidation of alcohol memories [5], and additional studies have shown that it also disrupted the reconsolidation of memories associated with fear [24] or with post-ingestive nutrients [25].

However, there is also evidence that some of the mechanisms underlying alcohol seeking may differ from those controlling natural reward seeking [5, 26–28]. Furthermore, there is evidence that memories for different rewards (including different drugs of abuse) are differentially processed [29–32]. Therefore, it is possible that alcohol memory reconsolidation is characterized by a unique transcriptional profile. As such, we sought to determine the transcriptional dynamics that underlie alcohol memory reconsolidation within the dorsal hippocampus (DH) and medial prefrontal cortex (mPFC) [5, 33, 34], brain regions implicated in alcohol use disorder [35, 36] and in the formation, retention and expression of drug memories [5, 37, 38].

## Results

### Alcohol memory reconsolidation depends on de novo gene transcription in the DH

While it has been established that the reconsolidation of alcohol memories requires de novo protein synthesis [5, 15], it remains unclear whether it is also dependent on de novo gene transcription. Therefore, we assessed the role of gene transcription during alcohol memory reconsolidation within the DH, a brain region implicated in alcohol use disorder [35] and involved in drug memory formation, retention, and expression [37, 38], in addition to memory reconsolidation [39, 40]. To form alcohol-associated memories, we employed the alcohol-conditioned place preference (CPP) paradigm. This paradigm has been used to examine the reinforcing properties of alcohol, as well as to explore the processing and maintenance of memories that evoke relapse to alcohol-seeking in rodents [41, 42], particularly in the DH [43].

**Fig. 1:**
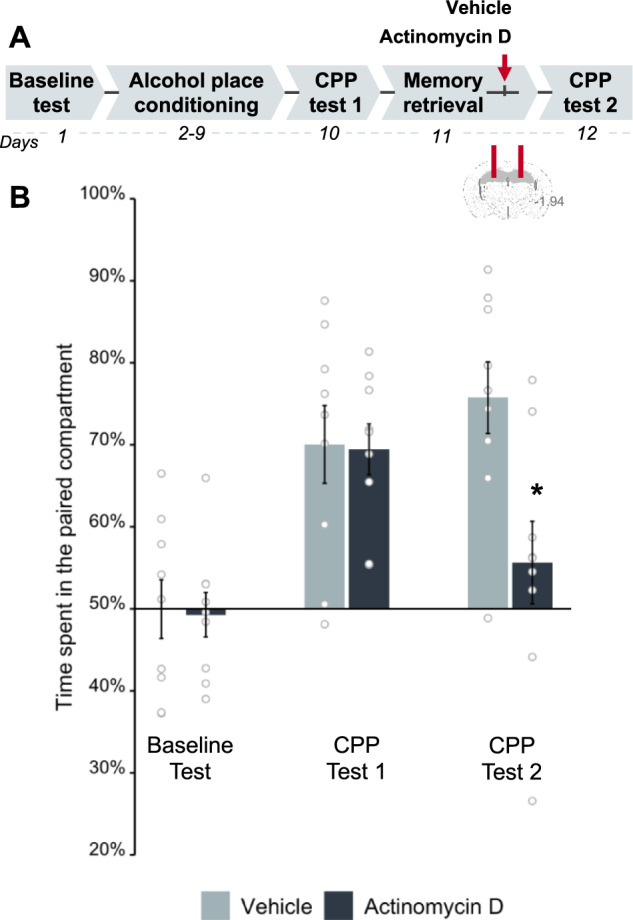
Inhibition of transcription in the dorsal hippocampus after alcohol memory retrieval disrupts the expression of alcohol-conditioned place preference (CPP). **A** Schematic illustration of the experimental design and timeline. Actinomycin D (4 µg/µl) was bilaterally infused into the dorsal hippocampus of mice immediately following the retrieval of alcohol memories. **B** Place preference scores, expressed as means ± S.E.M. of the percent of time spent in the alcohol-paired compartment. Mice that showed strong alcohol-CPP (t_(17)_ = 8.31, *p* < 0.0001) lost alcohol-place preference when memory retrieval was followed by intra-DH infusion of actinomycin D and not vehicle (mixed-model ANOVA: Test X Treatment (F_(1,16)_ = 9.97, *p* < 0.01), post hoc: CPP test 2 (*p* < 0.05)). *p < 0.05, **p < 0.05; *n* = 9 per group).

To assess the role of hippocampal gene transcription in alcohol memory reconsolidation, we formed alcohol-associated memories in the alcohol-CPP procedure, by conditioning one compartment of the CPP-apparatus to alcohol (Fig. 1A, experimental design). A day after confirming the strong preference for the alcohol-paired compartment in a CPP test, the mice were re-exposed to the alcohol-paired compartment for 3 min to retrieve alcohol-associated memories, as we previously demonstrated [44, 45]. Immediately after memory retrieval, actinomycin D (4 µg/µl; 0.5 µl per side) or vehicle were infused into the DH [19]. In a retention test conducted 24 h later, we found that mice that received post-retrieval actinomycin D did not show alcohol-CPP, whereas the preference for the alcohol-associated compartment remained high in the vehicle-treated mice (Fig. 1B; see Figure S1 for individual data). Thus, inhibition of gene transcription in the DH following memory retrieval led to the loss of alcohol-CPP, suggesting that the alcohol memory reconsolidation requires de novo gene transcription in the DH.

### Retrieval of alcohol-related memories causes a time-dependent upregulation of *Arc* and *Egr1* but not *Bdnf* mRNA expression in the DH and mPFC

**Fig. 2:**
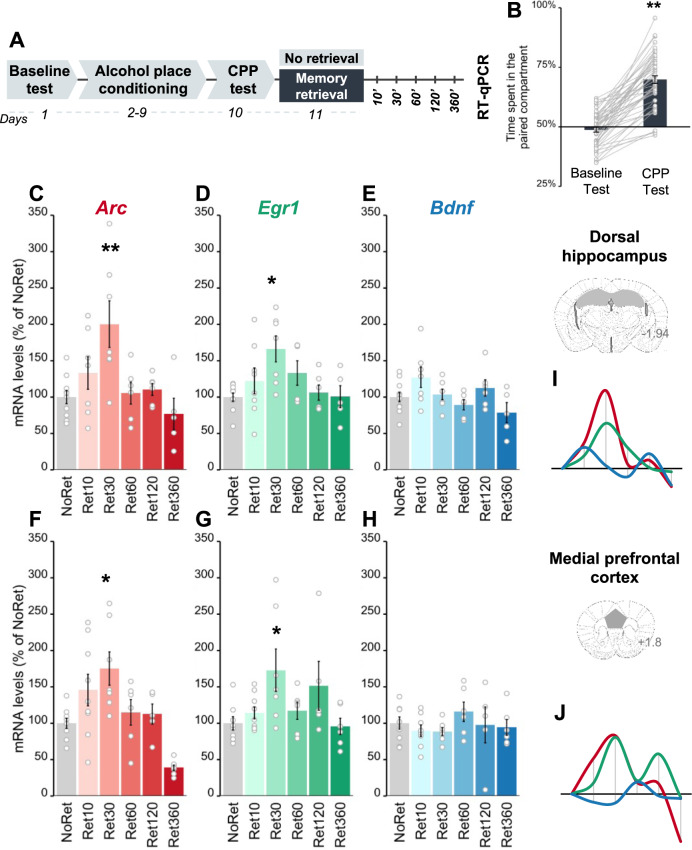
Alcohol memory retrieval triggers upregulation of *Arc* and *Egr1* but not *Bdnf* mRNA expression in the dorsal hippocampus and medial prefrontal cortex. **A** Schematic illustration of the experimental design and timeline. **B** Place preference scores, expressed as means ± S.E.M. of the percent of time spent in the alcohol-paired compartment (t_(47)_ = 13.82, *p* < 0.0001); **C**–**H** mRNA levels, normalized to *Gapdh*, of the percent of change from the control group (No Retrieval). qRT-PCR analysis revealed post-retrieval alterations in gene expression (one-way MANOVA; DH: Time (F_(15,86)_ = 2.42, *p* < 0.01); mPFC: Time (F_(15,86)_ = 2.96, *p* < 0.001): time-dependent upregulation of mRNA levels of *Arc* in the DH (NoRet vs Ret30’: *p* < 0.01) (**C**) and mPFC (NoRet vs Ret30’: *p* < 0.05) (**F**), of *Egr1* in the DH (NoRet vs Ret30’: *p* < 0.05) (**D**) and the mPFC (NoRet vs Ret30’: *p* < 0.05) (**G**), but not of *Bdnf* in the DH (**E**) or mPFC (**H**) (all *p*’s > 0.05); **I**, **J** Schematic representation of the time-dependent expression of *Arc* (red), *Egr1* (green), and *Bdnf* (blue) mRNA in the DH (**I**) and mPFC (**J**). Data are expressed as means ± S.E.M. *p < 0.05; **p < 0.01; *n* = 9–6 per group.

We next assessed whether alcohol memory retrieval alters the expression of the genes previously implicated in memory reconsolidation, namely activity‐regulated cytoskeleton‐associated protein (*Arc*) [5, 22, 46, 47], transcription factor *Egr1* (also known as *Zif268*) [19, 21, 48], and brain-derived neurotrophic factor (*Bdnf*) [49], in the DH and mPFC, brain regions implicated in the reconsolidation of drug memories [5, 33, 34, 39, 40, 50]. To assess *Arc*, *Egr1*, and *Bdnf* mRNA expression following alcohol memory retrieval, we first trained mice for alcohol-CPP (Fig. 2A, B). Twenty-four hours later, mice were re-exposed to the alcohol-paired compartment (Retrieval group) or were handled (No Retrieval group). We chose not to re-expose the control animals to the saline-paired context to prevent the retrieval of non-alcohol-related memories that are also characterized by changes in the expression of IEGs [46, 47, 50–52], or retrieval of a Pavlovian inhibitory alcohol memory, as the saline-paired compartment is associated with the absence of alcohol. Brain tissues were collected at five different time points after memory retrieval, and target mRNAs levels were analyzed.

Alcohol-memory retrieval triggered rapid but transient upregulation in the mRNA expression of *Arc* and *Egr1*, but not of *Bdnf*, in the DH (Fig. 2C–E, I). Specifically, *Arc* and *Egr1* mRNA levels peaked 30 min after alcohol memory retrieval, and returned to baseline levels within 60 min after memory retrieval, much like the No Retrieval group. In the mPFC, alcohol memory retrieval caused transient upregulation in *Arc* and *Egr1* but not *Bdnf* mRNA expression, similar to the expression pattern seen in the DH (Fig. 2G, H, J). The increases in *Arc* and *Egr1* mRNA expression in the DH and mPFC were preceded by increased phosphorylation of the transcription factor cAMP response element-binding protein (CREB) (Figure S2), previously shown to regulate the expression of these genes [53].

Together, the results show that the retrieval of alcohol-related memories induced a time-dependent upregulation in the expression of *Arc* and *Egr1* but not of *Bdnf* in the DH and mPFC, raising the possibility that altered expression of these genes may be involved in the reconsolidation of alcohol memories.

### The retrieval of alcohol-associated memories increases ARC protein levels in the DH

ARC has a well-established role in synaptic plasticity [54] and neuronal communication [55], and it was previously shown to play a role in the reconsolidation of various memories [5, 22, 46, 47]. Most relevantly, we previously showed that alcohol memory retrieval increased ARC protein levels in the amygdala and mPFC [5]. We now asked whether ARC protein levels were also increased in the DH, given the upregulation of *Arc* mRNA induced by alcohol memory retrieval (Fig. 2C). Accordingly, mice were trained to express alcohol-CPP, as described above (Figure S3A, B). A day after the CPP test, alcohol memories were retrieved, and brain tissues were collected 60, 120, or 360 min later. We found that ARC protein levels in the DH increased 60 min after alcohol memory retrieval, returning to baseline levels within the next hour (Figure S3). Together, these results suggest that alcohol memory retrieval increases both *Arc* mRNA and ARC protein expression in the DH.

### Downregulation of ARC expression in the DH disrupts alcohol memory reconsolidation

If the increase of ARC expression in the DH following alcohol memory retrieval is essential for alcohol memory reconsolidation, then its downregulation following alcohol memory retrieval should disrupt such memory, resulting in the abolition of alcohol-CPP expression. To downregulate ARC levels in the DH during alcohol memory reconsolidation, we used antisense oligodeoxynucleotides (AS-ODN) directed against *Arc* mRNA [56]. Knockdown of ARC in brain regions related to memory consolidation and reconsolidation using *Arc* AS-ODN was previously shown to disrupt the consolidation of aversive and appetitive memories [46, 47, 56, 57] and the reconsolidation of fear memories [46, 47, 57], as well as to impair morphine-associated memory reconsolidation [22].

**Fig. 3:**
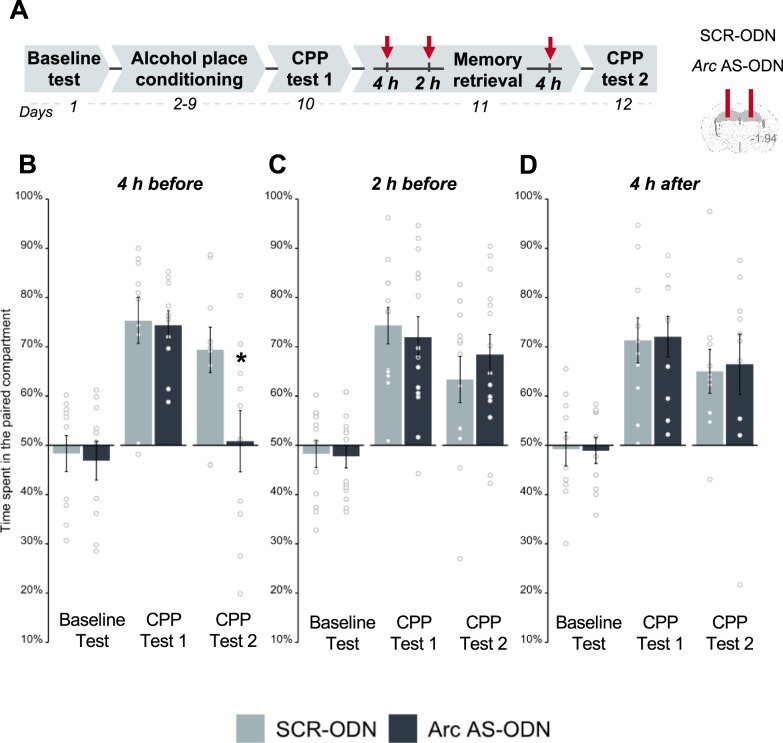
Downregulation of ARC protein expression in the dorsal hippocampus shortly after alcohol memory retrieval disrupts the expression of alcohol-conditioned place preference (CPP). **A**. Schematic illustration of the experimental design and timeline. Antisense oligodeoxynucleotides directed against *Arc* mRNA (*Arc* AS-ODN) or non-specific scrambled oligodeoxynucleotides (SCR-ODN) were infused into the dorsal hippocampus (DH) of mice at the indicated time points. **B**–**D** Place preference scores, expressed as means ± S.E.M. of the percent of time spent in the alcohol-paired compartment. Infusion of *Arc* AS-ODN disrupted the expression of alcohol-CPP when infused 4 h (mixed-model ANOVA; Test (F_(1,18)_ = 38.04, *p* < 0.001), and Test X Treatment (F_(1,18)_ = 13.48, *p* < 0.01); post hoc: CPP test 2 (*p* < 0.05)) (**B**) but not 2 h before memory retrieval (all *p*’s > 0.05) (**C**) or 4 h after memory retrieval (all *p*’s > 0.05) (**D**). *p < 0.05; *n* = 10–12 per group.

To test whether ARC downregulation disrupts alcohol memory reconsolidation and abolishes alcohol seeking, we trained mice to show alcohol-CPP (Fig. 3B, see Figure S4 for individuals’ data). A day after CPP test 1, the mice received an intra-hippocampal infusion of *Arc* AS-ODN or control scrambled (SCR)-ODN. Since the AS-ODN downregulated ARC protein levels 5 h after infusion [57] (Figure S5, AS-ODN validation), alcohol memory was retrieved 4 h after infusion, allowing the downregulation to occur an hour after retrieval, at around the peak of increase in ARC protein levels induced by alcohol memory retrieval (Figure S3C). When place preference was tested the next day, we found that alcohol-CPP was abolished in mice that had received *Arc* AS-ODN, whereas SCR ODN-treated mice still presented CPP (Fig. 3B). Our findings thus suggest that intra-hippocampal infusion of *Arc* AS-ODN disrupted the reconsolidation of alcohol memories by preventing the increases of ARC protein levels caused by memory retrieval.

To further test whether this memory disruption was due to blockade of the post-retrieval ARC induction specifically, we chose a second time point within the theoretical 5–6 h [9, 10] “reconsolidation window” for *Arc* AS-ODN infusion. Given that the increase in the expression of ARC protein peaked 1 h after memory retrieval and dropped back to baseline 2 h after memory retrieval (as indicated in Figure S3C), we targeted the ARC protein expression 3 h after memory retrieval. Thus, we infused *Arc* AS-ODN or control SCR-ODN into the DH 2 h before memory retrieval, which is expected to downregulate ARC levels 5 h later, i.e., 3 h after memory retrieval (Figure S5B). In a place preference test conducted a day later, we found that both groups persisted in showing alcohol-CPP (Fig. 3C). These findings indicate that downregulation of ARC levels past its retrieval-dependent induction does not interfere with the ongoing reconsolidation of alcohol memories. Moreover, here we demonstrate that the ARC-dependent “reconsolidation window” lasts no longer than 3 h after memory retrieval.

We further assumed that downregulation of ARC outside the “reconsolidation window”, i.e., more than 5–6 h after memory retrieval [9, 10], would not affect subsequent memory expression. To test this hypothesis, we infused *Arc* AS-ODN or control SCR-ODN into the DH 4 h after memory retrieval (Fig. 3D), which is expected to affect ARC protein expression 9 h after memory retrieval (i.e., 5 h later; Figure S5). Testing a day later revealed that both groups demonstrated strong preferences for the alcohol-paired compartment. These findings indicate that downregulation of ARC protein expression several hours after memory retrieval (i.e., outside the ARC-dependent reconsolidation window) does not affect the memories underlying the expression of alcohol-CPP.

Together, these findings suggest that the hippocampal upregulation of ARC protein expression observed shortly after alcohol memory retrieval is required for reconsolidating alcohol memories, as inhibition of these retrieval-induced increases of ARC protein levels led to the loss of alcohol seeking, likely by disrupting the memory reconsolidation process.

### Upon retrieval, appetitive alcohol- and non-alcohol-associated memories share similar *Arc* and *Egr1* transcriptional dynamics

**Fig. 4:**
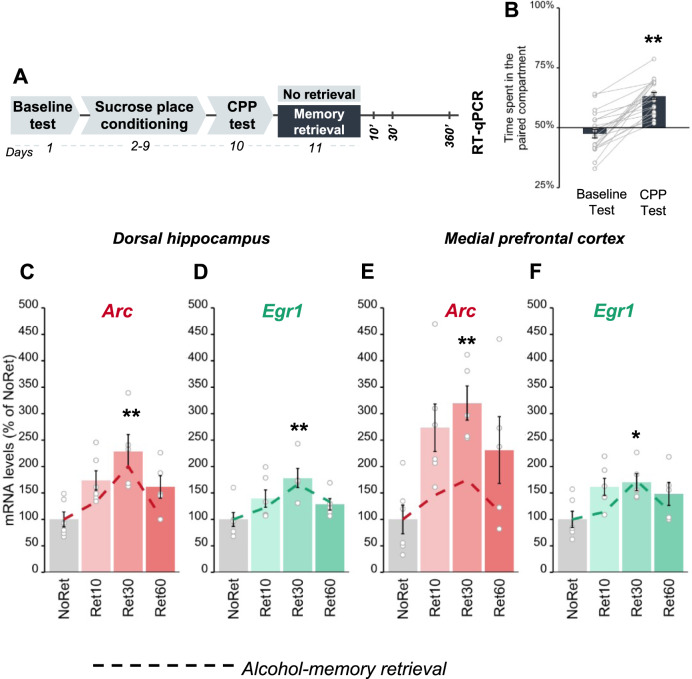
Sucrose memory retrieval triggers upregulation of *Arc* and *Egr1* in the dorsal hippocampus and medial prefrontal cortex. **A** Schematic illustration of the experimental design and timeline. **B** Place preference scores, expressed as means ± S.E.M. of the percent of time spent in the sucrose-paired compartment (t_(21)_ = 8.45, *p* < 0.0001); **C**–**F** mRNA levels, normalized to *Gapdh*, expressed as means ± S.E.M. of the percent of change, as compared with the control group (No Retrieval). qRT-PCR analysis revealed post-retrieval alterations in gene expression (one-way MANOVA; DH: Time (F_(6,34)_ = 2.82, *p* < 0.05); mPFC: Time (F_(6,34)_ = 2.13, *p* = 0.07)). *Arc* mRNA levels were transiently increased in the DH (NoRet vs Ret30’: *p* < 0.01) (**C**) and mPFC (NoRet vs Ret30’: *p* < 0.01) (**E**), while *Egr1* expression was increased in the DH (NoRet vs Ret30’: *p* < 0.01) (**D**) and the mPFC (NoRet vs Ret30’: *p* < 0.05) (**F**). mRNA expression levels after alcohol memory retrieval are shown as dashed lines. *p < 0.05; **p < 0.01; *n* = 9–6 per group.

Our findings implicating *Arc* and *Egr1* expression in the reconsolidation of alcohol memories are in line with previous studies showing these IEGs are implicated in the reconsolidation of different types of memory [19, 22, 46, 47, 57]. We assumed that the transcription and translation of these IEGs are not specific for alcohol, and rather may play a part in the common basic mechanisms for the processing of reactivated memories, including appetitive memories [16]. To further explore this possibility, we tested whether the retrieval of non-alcohol, sucrose-associated memories via a similar CPP protocol would alter *Arc* and/or *Egr1* mRNA expression, as it did with alcohol-related memories. For this, we first trained mice in a sucrose-CPP procedure similar to the alcohol-CPP procedure used above, pairing one compartment of the CPP apparatus with voluntary consumption of sucrose pellets (Fig. 4A, Experimental design). After four pairings, mice showed strong preference for the sucrose-associated compartment (Fig. 4B). Next, the sucrose-associated memory was retrieved by re-exposure to the sucrose-paired compartment. Brain tissues from the Retrieval and No retrieval control groups were collected 10, 30, or 60 min after memory retrieval. *Arc* and *Egr1* mRNA levels in the DH and mPFC were then assessed.

We found that sucrose memory retrieval caused rapid and transient upregulation of *Arc* and *Egr1* mRNA expression in both the DH and mPFC (Fig. 4C–F). As depicted by the dashed lines in Fig. 4C–F, the patterns of mRNA expression upregulation in both brain regions, induced by the retrieval of sucrose memories, resembled the upregulation of these genes observed upon alcohol memory retrieval. These findings thus suggest that rapid and transient upregulation in *Arc* and *Egr1* mRNA expression in the DH and mPFC is associated with the retrieval of both alcohol- and sucrose-associated memories.

### Characterization of alcohol-specific transcriptional dynamics for memory retrieval: a transcriptomic analysis

**Fig. 5:**
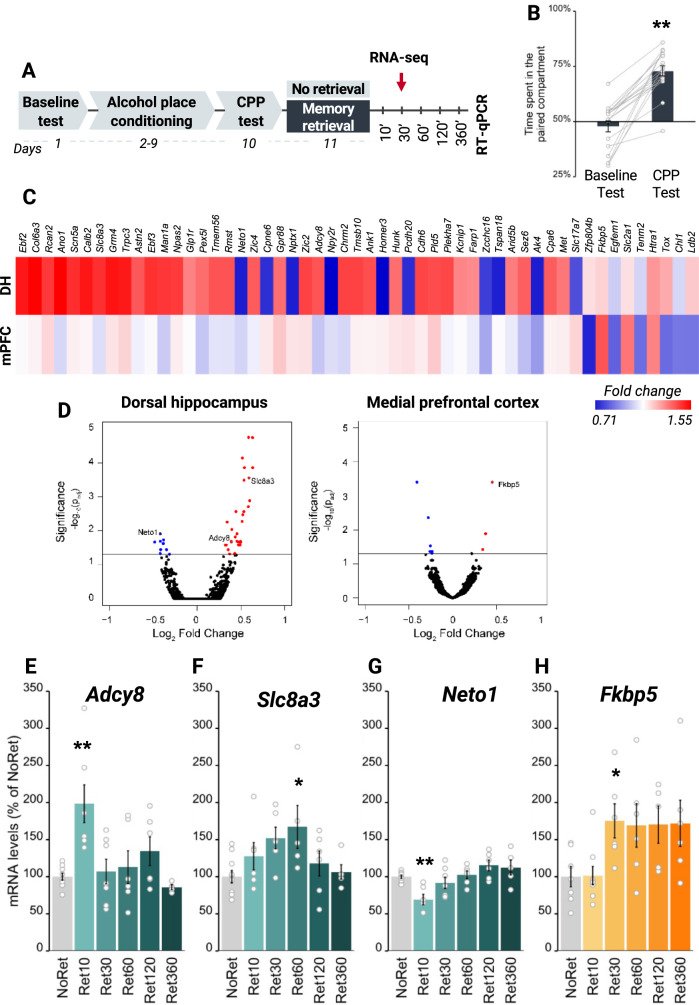
Alcohol memory retrieval alters transcriptomic dynamics in the dorsal hippocampus and medial prefrontal cortex. **A** Schematic illustration of the experimental design and timeline. **B** Place preference scores, expressed as means ± S.E.M. of the percent of time spent in the alcohol-paired compartment (t_(17)_ = 8.36, *p* < 0.0001); *n* = 8–9; **C** A heat map generated by hierarchical analysis of genes identified using DESeq2 shows significant changes in expression in the mPFC and/or DH following alcohol memory retrieval, as compared to the control No Retrieval group, with a significance cutoff-adjusted *p*-value (p_(adj)_ < 0.05). Red=upregulated genes with fold change of up to 1.55; blue=downregulated genes with fold change of down to 0.71. *n* = 2–3 biological replicates per condition, each replicate contained tissue from 3 mice pooled together. **D** A volcano plot provides an overview of the genes detected by RNA-sequencing. Log2-fold changes are plotted on the x-axis, and the negative log10 (*p*-value) is plotted on the y-axis. Differentially expressed genes appear above the line that indicates the significance threshold. Red=upregulated genes; blue=downregulated genes. **E**–**H** mRNA levels, normalized to *Gapdh*, expressed as means ± S.E.M. of the percent of change from the control group (No Retrieval). qRT-PCR analysis revealed time-dependent alternations in the levels of selected genes, detected via RNA-sequencing. In the DH: *Adcy8* (one-way ANOVA; Time (F_(5,33)_ = 5.08, *p* < 0.01*)*; post hoc: NoRet vs Ret10 (*p* < 0.01)) (**E**), *Slc8a3* (Time (F_(5,33)_ = 2.78, *p* < 0.05); post hoc: NoRet vs Ret60 (*p* < 0.05)) (**F**), *Neto1* (Time (F_(5,33)_ = 6.27, *p* < 0.01); post hoc: NoRet vs Ret10 (*p* < 0.01)) (**G**). In the mPFC: *Fkbp5* (Time (F_(5,33)_ = 3.07, *p* < 0.05); post hoc: NoRet vs Ret30 (*p* < 0.05)) (**H**). *p < 0.05, **p < 0.01; *n* = 10*–*6.

Given our finding that *Arc* and *Egr1* transcription were altered upon retrieving both alcohol- and non-alcohol-associated memories, we next sought to identify the transcriptomic signature specific for alcohol memory retrieval by performing RNA-seq analysis of the DH and mPFC (Fig. 5A, Experimental design). To this end, following alcohol-CPP training (Fig. 5B), alcohol-associated memories were retrieved (with a No retrieval control group). The DH and mPFC were collected 30 min later and processed for RNA-seq analysis (Fig. 5C, D).

Using DESeq2 [58], we identified a set of 44 genes whose levels of expression were significantly altered in the DH, with 34 genes being upregulated, and 10 genes being downregulated (Padj < 0.05; Fig. 5C, D and Table S2). We further found that in the mPFC, the expression of 9 genes was significantly altered (3 were upregulated and 6 were downregulated); none of these genes overlapped with those DH genes showing altered expression (Fig. 5C). We then focused on the genes that were previously implicated in memory or/and addiction studies. Thus, we evaluated the post-retrieval expression changes in the genes of interest detected by RNA-seq by performing quantitative reverse transcriptase-polymerase chain reaction (qRT-PCR) analysis of brain samples collected from a different batch of animals at five time points following alcohol-memory retrieval. Overall, we tested 10 genes in the DH and 2 genes in the mPFC (Table S2).

We found that alcohol-memory retrieval led to downregulation of the mRNA expression of *Adcy8* (encoding adenylate cyclase 8) and *Slc8a3* (encoding solute carrier family 8 (sodium/calcium exchanger), member 3), and to downregulation of *Neto1* (encoding neuropilin (NRP) and tolloid (TLL)-like 1) expression in the DH (Fig. 5E–G), as well as to upregulation of *Fkbp5* expression (encoding FK506 binding protein 5) in the mPFC. Consistent with our earlier qRT-PCR results (Fig. 2), *Arc* and *Egr1* expression showed a trend of upregulation by memory retrieval in the DH and mPFC also in the RNA-seq (Table S2). Finally, highly similar results were obtained using a newer reference genome, mm10 (Figure S6).

**Fig. 6:**
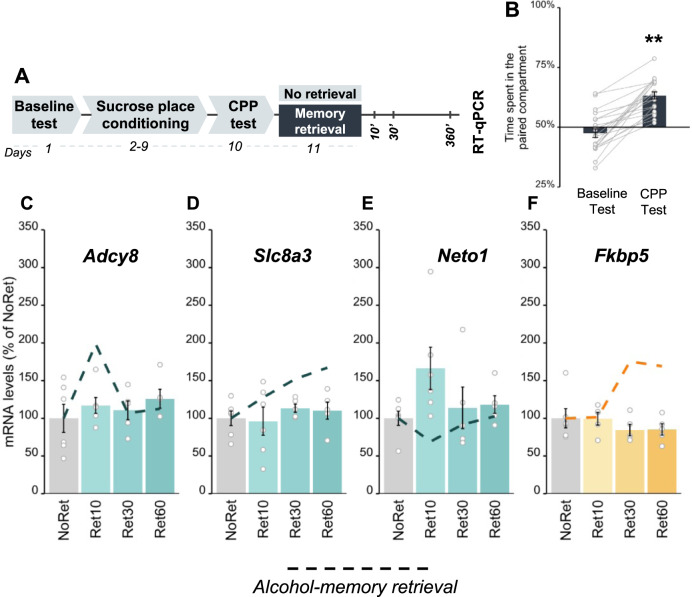
Sucrose memory retrieval does not alter the expression of *Adcy8*, *Slc8a3*, or *Neto1* in the dorsal hippocampus or of *Fkbp5* in the medial prefrontal cortex. **A** Schematic illustration of the experimental design and timeline. **B** Place preference scores, expressed as means ± S.E.M. of the percent of time spent in the sucrose-paired compartment (t_(21)_ = 8.45, *p* < 0.0001); **C**–**F** mRNA levels, normalized to *Gapdh*, expressed as means ± S.E.M. of the percent of change from the control group (No Retrieval). qRT-PCR analysis did not reveal differences in the mRNA levels of *Adcy8, Slc8a3,* or *Neto1* (**A**–**C**), and *Fkbp5* (**D**) in the DH and mPFC, respectively, between the Retrieval and No retrieval groups (one-way ANOVA; all *p*’s > 0.05). mRNA expression levels after alcohol memory retrieval (presented in Fig. 5E–H)) are shown as dashed lines; **p < 0.01; *n* = 9–6 per group.

We next tested whether the expression of specific genes we found to be altered upon retrieval of alcohol memories were affected by the retrieval of non-alcohol, sucrose-associated memories, in a manner similar to the common upregulation of *Arc* and *Egr1* mRNA expression (Fig. 6A, B). As shown in Fig. 6C–F, *Adcy8, Slc8a3, Neto1*, and *Fkbp5* mRNA expression was not affected by sucrose memory retrieval, although a trend towards increased expression of *Neto1* mRNA was noted after 10 min of sucrose memory retrieval, i.e., in the opposite direction to the decreased mRNA expression induced by alcohol memory retrieval.

To summarize, these experiments identified a unique transcriptional dynamics triggered by alcohol memory retrieval, which is not common for memories of a natural reward, namely, sucrose-associated memories.

## Discussion

We show here that the reconsolidation of alcohol-associated memories requires de novo gene transcription, and that alcohol seeking can be disrupted by inhibiting transcription following alcohol memory retrieval. Importantly, our findings suggest that while the altered expression of some genes is likely a common mechanism for the reconsolidation of several types of memories, the processing of alcohol-related memories is also characterized by a unique transcriptional profile.

We found that the retrieval of either alcohol memories or of memories associated with a natural reward (sucrose) triggered similar increases in mRNA expression of the IEGs *Arc* and *Egr1* in the DH and mPFC. In contrast, RNA-seq analysis revealed a subset of genes (*Adcy8*, *Slc8a3*, and *Neto1* in the DH, and *Fkbp5* in the mPFC) of which expression was altered selectively by the retrieval of alcohol, but not by sucrose reward memories, raising the intriguing possibility that alcohol-associated memories that trigger relapse have unique molecular mechanisms that could be targeted to disrupt them selectively.

We show that the downregulation of ARC shortly after alcohol memory retrieval abolishes alcohol seeking (the expression of alcohol CPP), indicating a critical role for hippocampal ARC expression in the reconsolidation of alcohol memories. *Arc* is a CREB-regulated IEG, rapidly induced by neuronal activity, and known to regulate synaptic plasticity and mediate memory formation [56]. We previously showed that inhibition of the translational machinery that controls ARC protein synthesis disrupted alcohol memory reconsolidation and prevented relapse to alcohol seeking and drinking in a rat self-administration paradigm [5]. Indeed, *Arc* has been described as a key player in the reconsolidation of drug [5, 13, 22]- and fear [46, 47]-associated memories.

We found that *Arc* mRNA expression peaked 30 min after alcohol memory retrieval, and returned to baseline levels within the next 30 min, followed by a transient increase in ARC protein levels, peaking 1 h after memory retrieval. These transient ARC dynamics imply that alcohol memory retrieval induces rapid mRNA [59] and protein [60] degradation. Accordingly, we found that alcohol memory reconsolidation was disrupted when we knocked-down the expression of ARC 1 h but not 3 or 9 h after memory retrieval. It is accepted that the “reconsolidation window” lasts ~5–6 h, as manipulations conducted 5–6 h after memory retrieval failed to affect targeted behaviors [7, 10, 11]. Our results, therefore, suggest that the reconsolidation window might be in fact narrower than 5 h, at least when ARC protein expression upregulation is required for memory re-stabilization. This finding emphasizes the idea that the duration of the post-retrieval memory lability varies in the experimental settings depending on the manipulations and molecular event of interest.

While we localized the causal role of ARC in alcohol memory reconsolidation to the DH, a brain region previously implicated in the reconsolidation of contextual drug memories [39, 40, 48, 50], *Arc* and *Egr1* mRNA expression was also upregulated in the mPFC. Both the prelimbic and infralimbic prefrontal subregions have been implicated in drug memory reconsolidation [33, 34], suggesting the mPFC to be a candidate brain region that regulates alcohol seeking via reconsolidation mechanisms. Our current finding is consistent with our previous observation of upregulated ARC protein levels in this brain region following alcohol memory retrieval in an operant alcohol self-administration procedure [5].

In addition to *Arc*, we found that the mRNA levels of *Egr1*, but not *Bdnf*, were increased in the DH and mPFC by alcohol memory retrieval. Increased *Egr1* mRNA expression has been previously implicated in the reconsolidation of fear and drug memories [19, 21, 48, 61], raising the possibility that this transcription factor also plays a role in the reconsolidation of alcohol memories. Whereas the role of *Bdnf* in memory reconsolidation remains controversial [19, 49], *Bdnf* induction is known to be crucial for memory acquisition and extinction learning [19, 62]. Thus, the lack of change in *Bdnf* expression in our study could suggest that our memory retrieval procedure did not initiate extinction learning in parallel with memory retrieval. Moreover, we recently reported that although the mRNA levels of *Bdnf* in the DH are not increased by alcohol memory retrieval per se, the expression of the growth factor were elevated when the retrieval is followed by aversive counterconditioning that prevented relapse [44], suggesting a complex role of *Bdnf* in alcohol memory dynamics.

Our findings indicate that the increases in the IEGs expression are not unique to the reconsolidation of alcohol-related memories. Indeed, ARC and EGR1 were previously implicated in the reconsolidation of several types of memories, including fear memories [19, 46, 47, 62], recognition memories [21], and memories associated with different drugs of abuse [20, 22, 61]. However, there is also growing evidence that some of the mechanisms underlying alcohol seeking may differ from those controlling natural reward seeking [5, 27, 28, 63]. Also, there is evidence that memories for different rewards (and for different drugs, in particular) are differentially processed [29–32]. Our RNA-seq analysis findings indeed revealed the unique transcriptomic signature induced by alcohol memory retrieval. Specifically, we found 44 genes in the DH and a different set of 9 genes in the mPFC, which showed significant changes following alcohol memory retrieval. Following further data validation, we focused on one gene in the mPFC (*Fkbp5*) and three genes in the DH (*Adcy8, Scl8a3,* and *Neto1*) and found that their expression was not affected by the retrieval of sucrose-associated memories. This suggests that unlike *Arc* and *Egr1*, these 4 specific genes likely do not play general roles in the processing of reward memories, yet may rather play selective roles in the processing of alcohol memories.

Interestingly, these four genes were previously implicated in alcohol use disorder and in learning and memory. Thus, *Fkbp5* encodes the FK506 Binding Protein 5, a regulator of the stress-neuroendocrine system [64]. *FKBP5* variants modulate the severity of alcohol withdrawal syndrome [65], and predict the propensity of heavy drinking in humans [66–68]. Deletion of *Fkbp5* was shown to increase alcohol withdrawal severity [65] and alcohol drinking [67] in mice, whereas pharmacological inhibition of the protein reduced moderate alcohol consumption, and reinstatement of CPP in mice [69] and reduced alcohol drinking in stressed male rats [70]. The levels of *Adcy8* mRNA, encoding adenylate cyclase 8 (AC8) that catalyzes cAMP formation in response to calcium influx and recently marked as a possible regulator of alcohol intake [28, 71], were decreased in blood cells from long-term abstinent alcoholics [72]. Deletion of *Adcy8* was previously shown to reduce alcohol drinking and increase sensitivity to the sedative effects of alcohol in mice [73]. In addition, chronic alcohol exposure in rats upregulated the brain expression of solute carrier family 8 (sodium/calcium exchanger), member 3 (NCX3), the protein encoded by *Scl8a3* [74, 75]. Alcohol consumption in rats was also associated with reduced brainstem expression of *Neto1* [76], encoding neuropilin tolloid-like 1 (NRP1), a component of the NMDA-receptor complex [77]. This protein is involved in synaptic reorganization and transmission in the hippocampus [78], and is required for spatial learning and memory [77]. Thus, our findings show that the mere retrieval of alcohol memories, even without any pharmacological effects of alcohol, affect the expression of these genes. However, their specific role in alcohol memory reconsolidation and relapse remains to be tested.

A limitation of the present study is that the RNA-seq assay was conducted on the bulk mPFC and DH tissues, rather than on individual cells activated by memory retrieval. Specifically, recent studies have suggested that memories in general [79, 80], and specifically alcohol-related memories [32, 81, 82], are encoded via the activation of neuronal ensembles composed of a small number of neurons, which can be identified via neuronal activation markers (e.g., cFOS) [80]. Indeed, similar IEG (cFOS) activation patterns by retrieval of operant-conditioned alcohol and sweet reward memories were found in the mPFC and additional mesolimbic regions in the rat brain [32, 81]. Thus, it is possible that conducting the RNA-seq assay selectively on the neuronal ensemble activated by memory retrieval would have yielded more accurate results.

In summary, our findings suggest that alcohol memory retrieval induces two parallel transcription programs. One program, conveyed via common molecular mechanisms of learning and memory, including the IEGs *Arc* and *Egr1*, is engaged in the reconsolidation of memories, in general. The other transcription program, launched by alcohol memory retrieval, is controlled by genes that specifically promote alcohol-related behaviors. This dual-processing model for alcohol memories raise the possibility that memory reconsolidation for different memories may have similar dual-processing molecular mechanisms, with some components shared by multiple memories, and others unique to each memory type. This hypothetic model remains to be assessed by testing whether manipulating these genes affects alcohol- and sucrose-memory reconsolidation differentially. If so, it would suggest that it may be possible to aim at reward-specific molecular targets to treat disorders related to pathogenic memories, such as addiction, rather than disrupt major molecular mechanisms essential for many functions beyond alcohol memory reconsolidation.

## Materials and methods

See Supplementary Information for details on the apparatus, drugs and reagents, oligodeoxynucleotide (ODN) design and preparation, western blot and qRT-PCR analyses and RNA-seq library preparation.

### Animals

Male and female C57BL/JRccHsd mice (25–30 g), housed 3–4/cage were bred at the Tel-Aviv University Animal Facility (Israel), and kept under a 12 h light-dark cycle (lights on at 7 a.m.), with food and water available ad libitum. In experiments involving sucrose place-conditioning, access to food was restricted for 6 h prior training sessions to boost the mice’ motivation to collect sucrose pellets. This short duration of food deprivation is based on findings that in the daytime, a 2–6 h fasting does not affect feeding behavior [83], and does not trigger a significant shift in metabolic markers [84], thus minimizing effects of the food restriction per se. Mice were weighed twice a week to control for weight loss. All experimental protocols were approved by and conformed to the guidelines of the Institutional Animal Care and Use Committee of Tel Aviv University, and to NIH guidelines of the (animal welfare assurance number A5010-01). All efforts were made to minimize the number of animals used.

### Behavioral procedures

#### Place conditioning

##### Alcohol-conditioned place preference (CPP)

All mice were habituated to daily i.p. saline injections for 3 days.

*Baseline test (day 1)*: On the first day, the sliding door was retracted and mice were allowed to freely explore the entire apparatus for 30 min.

*Alcohol place conditioning (days 2–9)*: Training started 24 h after the baseline test with one session administered per day over 8 days, with the sliding door closed. On days 3, 5, 7, and 9, the mice received alcohol (1.8 g/kg, 20% v/v; i.p.) and immediately confined to the paired compartment for 5 min. This dose and conditioning duration were previously shown to produce alcohol-induced CPP [42, 85]. On the alternate days (i.e., days 2, 4, 6, and 8), the mice received saline solution and were confined to the unpaired compartment for the same duration as on the alcohol-conditioning day. Paired compartments were counterbalanced.

*Place preference test 1 (day 10)*: Place preference testing was as described for the baseline test, and served to index alcohol-CPP [41]. Preference was defined as an increase in the percent of time spent in the alcohol-paired compartment during place preference test 1, as compared to the baseline test.

*Memory retrieval (day 11)*: Prior to this stage, the mice were assigned to different experimental conditions (matched for CPP scores and sex). During a memory retrieval session, the mice were confined to the alcohol-paired compartment for 3 min, and then returned to their home cages. Control mice were handled briefly.

*Place preference test 2 (day 12)*: The mice were subjected to a place preference test identical to place preference test 1.

##### Sucrose CPP

*Sucrose pre-training (days 1–6)*: On days 1–3, the mice were habituated to collect 5–6 sucrose pellets (45 mg, Dustless Precision Pellets, Bio-Serv, Frenchtown, NJ, USA) in home cages. On days 4–6, mice were trained to collect 3 pellets in an empty Plexiglas box during 30 min sessions. Mice that collected fewer than six pellets (out of a total of nine pellets over the 3 days) during sucrose training were excluded from the experiment (2 mice out of 32).

*Baseline test (day 7)*: On the seventh day, the sliding door in a CPP-chamber was retracted and the mice were allowed to explore the entire apparatus for 30 min. An unbiased apparatus/unbiased assignment approach, as described in the alcohol-CPP procedure, was also adopted here.

*Sucrose place-conditioning (days 8–15)*: Training started 24 h after the baseline test with one session per day over 8 days, with the sliding door closed. On days 9, 11, 13, and 15, mice were placed in the paired compartment, and a min later, three sucrose pellets were scattered on the floor. Mice were returned to their home cages 15 min later. On the alternate days (i.e., days 8, 10, 12, and 14), mice were placed in the unpaired compartment for 15 min with no interference. Paired compartments were counterbalanced.

*Place preference test 1 (day 16)*: Place preference testing was as described for the baseline test, and served to index sucrose-CPP. Preference was defined as an increase in the percent of time spent in the sucrose-paired compartment during place preference test 1, as compared to the baseline test.

*Memory retrieval (days 17)*: The sucrose memory retrieval procedure was identical to the alcohol memory retrieval procedure (see above).

### Oligodeoxynucleotide (ODN) validation

*Arc* antisense ODN (AS-ODN) and scrambled ODN (SCR-ODN) (Sigma-Aldrich, Rehovot, Israel) design followed the guidelines described previously [56, 86].

#### *Arc* AS-ODN functional validation

*Arc* AS-ODN (2 nmol/µl, 0.5 µl, 0.25 µl/min) were infused into the DH in one hemisphere, and SCR-ODN (2 nmol/µl, 0.5 µl, 0.25 µl/min) was infused into the other hemisphere (the sides were counterbalanced). Two or four hours later, treated mice explored an unfamiliar context (CPP compartment, see Apparatus description, above) for 5 min to induce novelty-dependent ARC expression. Mice were euthanized an hour later (i.e., 3 or 5 h after ODN infusion), brain tissues were collected, and ARC protein levels were assessed by Western blot analysis (Figure S4).

### Surgery and intra-hippocampal microinfusion

Surgery and microinfusions were conducted as described previously [42, 87].

#### Surgery

Stereotaxic surgeries were conducted under isoflurane anesthesia. Mice were placed in a stereotaxic frame (RWD Life Science, Shenzhen, China) and bilateral guide cannulae (C235G-2.6, 26G; Plastics One Inc., Roanoke, VA, USA) were aimed at the DH at the following coordinates (−2 mm posterior to bregma, ±1.3 mm mediolateral, −1.45 mm ventral to the skull surface). Cannulae were secured with dental acrylic. Matching dummy cannulae (Plastics One Inc., Roanoke, VA, USA) were inserted into the guide cannulae and topped with dust cups to keep the injector site covered and clear of debris. The mice were allowed to recover for 7–10 d prior to alcohol-CPP training.

#### Intra-hippocampal infusions

Actinomycin D (4 µg/µl, 0.5 µl per side, 0.25 µl/min, in DMSO) [19] or an equivalent volume of vehicle was microinjected into the DH immediately after memory retrieval. Infusion of actinomycin D into the hippocampus at a similar concentration was shown to disrupt memory reconsolidation [19]. *Arc* AS-ODN or control SCR-ODN (2 nmol/µl, 0.5 µl/side, 0.25 µl/min; in PBS) was microinjected into the DH 4 h or 2 h before or 4 h after memory retrieval. After removal of the dust cup and dummy cannulae, microinfusion was conducted over 2 min to awake gently restrained mice, using injection cannulae (33 G; Plastics One Inc., Roanoke, VA, USA) extending 0.5 mm beyond the guide cannula tip. Injection cannulae were left in place for an additional 2 min. After infusion, dummy cannulae were inserted into the guide cannulae, secured with the dust cup, and the animals returned to their home cages. Cannulae locations were verified in 30 μm-thick coronal sections of paraformaldehyde-fixed tissue stained with cresyl violet.

### Data analysis

Place preference was assessed as the percentage of time spent in the alcohol/sucrose-paired compartment, relative to the total test time. Mice that spent >75% of time in either of the compartments during Baseline test were excluded from the study (9 out of 267). CPP establishment was confirmed by comparing CPP scores between the baseline and CPP test 1, and was analyzed by a paired t-test. Since the expression of CPP (i.e., memory formation) is required for memory retrieval, data from mice that did not show CPP (a minimum 5% increase in preference between baseline and Test 1) were excluded from the experiment (48 out of 258).

Alcohol-CPP following interference with memory reconsolidation was analyzed by a mixed-model ANOVA with a between-subjects factor of Treatment (Actinomycin D or ODN) and a within-subjects factor of Test (CPP test 1, CPP test 2). Significant interactions were analyzed by a Student–Newman–Keuls post hoc test. The densities of Western blot immunoreactive ARC protein levels were normalized to those GAPDH and analyzed by a one-way ANOVA with a between-subjects factor of Treatment, followed by Dunnett’s post hoc test. qRT-PCR mRNA expression data were normalized to *Gapdh*, and expressed as percentage of expression of a control group. Data from all genes for each brain region were analyzed by a one-way MANOVA with a between-subjects factor of Treatment, followed by Dunnett’s test for each gene, for comparison to the data obtained from a control (No Retrieval) group.

For RNA-seq, we pooled tissues from 3 animals for each replicate and prepared the following number of replicates: 3 for the dorsal hippocampus (DH)—no retrieval, 2 for DH—retrieval, 3 for the prefrontal cortex (PFC)—no retrieval, and 2 for (PFC)—retrieval. After total RNA isolation with Trizol, sequencing libraries were generated using the method we developed [88]. The libraries were subjected to 50 bp single-end sequencing with Illumina Hiseq 2000. After confirming the quality of sequencing data by FastQC, reads were mapped to the mm9 reference genome using Bowtie2 [89] and annotated with Tophat2 [90]. Reads mapped to genes were quantified by FeatureCounts [91]. We excluded *Rn45s*, *Lars2*, *Rn4.5s*, *Cdk8*, *Zc3h7a* and the mitochondrial chromosome to avoid counts of over-amplified genes that could skew library normalization as previously described [92]. DESeq2 [58] was used to identify differentially expressed genes (DEG) in the Retrieval group, compared to the No Retrieval control group, with a significance cutoff-adjusted *p*-value (p_(adj)_ < 0.05).

## Supplementary information

Supplementary information, tables and figures

## Data Availability

RNA-seq data are available at NCBI Gene Expression Omnibus at https://www.ncbi.nlm.nih.gov/geo/query/acc.cgi (GSE205586) with a reviewer token: knyfwwiorbutvuv.
