# Supplementary material for: Alcohol-specific transcriptional dynamics of memory reconsolidation and relapse
Source: Transl Psychiatry. 2023 Feb 15;13:55. doi: 10.1038/s41398-023-02352-2 (PMC9932068; doi:10.1038/s41398-023-02352-2)
Supplement: Supplementary file 1 — Supplementary information, tables and figures [file 41398_2023_2352_MOESM1_ESM.pdf]

Supplementary Information – Materials, Methods, Tables, Figures

Apparatus

Place conditioning

All place conditioning experiments were performed in open ceiling Plexiglas boxes (30 x 30 x 20 cm) divided into two equal-sized compartments by a sliding door. The compartments differed from each other in terms of wall pattern (horizontal vs. vertical black and white stripes, 1 cm-wide) and floor surface (white textured plastic with bulging circles vs. bulging stripes). The horizontal striped pattern on the walls was always matched with the bulging circles on the floor, whereas the vertical stripes were linked with the bulging stripes. Animals that spent >75% of time in either compartment during the Baseline Test were excluded from the study. This allowed the use of an unbiased design, in which the two compartments were equally preferred before conditioning, as indicated by the group average (unbiased apparatus), and pseudo-randomly assigned to the experimental conditions (unbiased assignment procedure)[31]. Each place-conditioning chamber was assigned to a single sex and placed in a sound-attenuating chamber equipped with a LED light stripe on the walls and a ceiling camera that registered mouse behavior. Data were recorded with an Ethovision XT 11.5 video tracking system (Noldus, Wageningen, Netherlands).

Drugs and reagents

Ethanol absolute (Bio-Lab, Jerusalem, Israel) was diluted in sterile saline solution (0.9 M NaCl) for systemic injections. Actinomycin D was purchased from Sigma-Aldrich (Rehovot, Israel) and dissolved in DMSO (Sigma-Aldrich, Rehovot, Israel)). Isoflurane was obtained from Piramal Critical Care (Bethlehem, PA, USA). Fast SYBR Green Master Mix, TRIzol reagent and RevertAid kit were supplied by Thermo-Fisher Scientific (Waltham, MA, USA). DNA oligonucleotides (RT-qPCR primers) were obtained from Sigma-Aldrich (Rehovot,

Israel). Mouse monoclonal antibodies against 1:250 anti-pCREB ARC (sc-17839) and GAPDH (sc-32233) were purchased from Santa Cruz Biotechnology (Santa Cruz, CA, USA). Nitrocellulose membranes were purchased from Millipore (Billerica, MA, USA). Protease and Phosphatase Inhibitor Cocktail (100x) and enhanced chemiluminescent (ECL)-horseradish peroxidase (HRP)HRP substrate were purchased from Thermo Fisher Scientific (Kiryat Shmona, Israel).

Oligodeoxynucleotide (ODN) design and preparation

The *Arc* AS-ODN encoded an antisense sequence for the *Arc*/*Arg3.1* mRNA sequence that overlaps the translation start site, and was previously shown to knockdown *Arc* mRNA translation in mice[70]. In addition, it was reported to inhibit ARC protein expression in the hippocampus without affecting the translation of other genes[42,70] and to impair the reconsolidation of fear memories[36]. In a pilot study, we confirmed the ability of *Arc* AS-ODN to reduce ARC protein expression in the DH, as compared to a SCR-ODN control (~40% reduction) (Figure S5). The SCR-ODN, containing the same nucleotides as the *Arc* AS-ODN but assembled in a random sequence did not show significant homology to sequences in the GenBank database and served as a control. Both ODNs contained phosphorothioate linkages at the bases of both the 5′ and 3′ ends and phosphodiester internal bonds, given how this nucleotide design was reported to be more stable than a unmodified phosphodiester ODN *in vivo* and less toxic than a fully phosphorothioated ODN[42]. The following sequences were used (* denotes a phosphorothioate linkage): 5′-G*T*C*CAGCTCCATCTGGT*C*G*T-3′ (*Arc* AS-ODN) and 5′- C*G*T*GCACCTCTCGCAGG*T*T*T-3′ (SCR-ODN).

Western blot analysis

Western blot analysis was conducted as we previously described[5]. Briefly, snap-frozen samples were homogenized in buffer containing (in mM): 25 Tris-HCl, pH 7.6, 150 NaCl, 1 EDTA, 1% (v/v) NP-40, 0.5% (w/v) sodium deoxycholate, 0.1% (w/v) sodium dodecyl sulfate (SDS) and

protease and phosphatase inhibitors. Protein concentrations were determined using a BCA assay, and equal amounts of each sample (40 µg) was denatured with Laemmli buffer, resolved by 10% SDS–polyacrylamide gel electrophoresis (SDS-PAGE) and electro-transferred to a nitrocellulose membrane. Membranes were blocked for 1 hour at room temperature in 5% (w/v) BSA in Tris-buffered saline and 0.1% (v/v) Tween 20 (TBST) and then incubated overnight at 4°C with primary antibodies (anti-pCREB 1:250; anti-ARC 1:500). After washing with TBST, the membranes were incubated for 1 h at room temperature with horseradish peroxidase (HRP)-conjugated secondary antibodies (1:5000). To visualize the bound antibodies, we used ECL substrate for HRP on ImageQuant LAS 500 imager system. Membranes were then stripped for 30 min at 50°C in buffer containing 100 mM 2-Mercaptoethanol, 2% (w/v) SDS and 62.5 mM Tris-HCl, pH 6.7, followed by extensive washing in TBST before re-blocking and re-probing with tCREB (1:500 anti-CREB) and/or GAPDH-specific antibodies. The optical densities of the relevant immunoreactive bands were quantified using ImageLab software (version 4.1, Bio-Rad). The optical density values of the ARC protein immunoreactive bands were normalized to those of GAPDH. Results are expressed as a percentage of the values obtained with a control group.

Quantitative reverse transcriptase polymerase chain reaction (qRT-PCR)

Following brain dissection, tissue samples were immediately snap-frozen in liquid nitrogen and stored at −80°C until use. Frozen tissues were mechanically homogenized in TRIzol reagent and total RNA was isolated from each sample according to the manufacturer's recommended protocol. mRNA was reverse transcribed to cDNA using the Reverse Transcription System and RevertAid kit. Plates (96 wells) were prepared for SYBR Green cDNA analysis using Fast SYBR Master Mix. Samples were analyzed in triplicate/duplicate with a Real-Time PCR System (StepOnePlus, Applied Biosystems), and quantified against an internal control gene *Gapdh*. We used the following reaction primers sequences: *Arc* forward,

5′- ACCGGGGGTCACTAAGTATGG -3′; reverse, 5′- CATTCTCCTGGCTCTGTAGGC -3′; *Egr1*(*Zif268*) forward, 5′- TGAGCACCTGACCACAGAGTC -3′; reverse, 5′- TAACTCGTCTCCACCATCGC-3′; *Bdnf IV* forward, 5′- GCAGCTGCCTTGATGTTTAC -3′; reverse, 5′- CCGTGGACGTTTACTTCTTTC -3′; *Gapdh* forward, 5′- CCAGAACATCATCCCTGC-3′; reverse, 5′- GGAAGGCCATGCCAGTGAGC-3′. Thermal cycling was initiated with incubation at 95°C for 20 s (for SYBR Green activation), followed by 40 cycles of PCR with the following conditions: Heating at 95°C for 3 s and then 30 s at 60°C. Relative quantification was calculated using the ΔΔCt method. The expression of target genes was normalized to that of *Gapdh*, and is expressed as percentage of the control group.

RNA-seq library preparation

Thirty minutes after memory retrieval or handling, mice were euthanized by cervical dislocation. Following brain dissection, tissue samples were immediately snap-frozen in liquid nitrogen and stored at −80°C until use. Frozen brain tissue was re-suspended in 0.6 mL TRI Reagent (Sigma T9424) and Dounce-homogenized for 40 strokes using tight pestle. RNA was purified using phenol-chloroform extraction and isopropanol precipitation and quality was assessed on an agarose gel. A 2 μg aliquot of total RNA from each sample was depleted of ribosomal RNAs using a Ribominus Eukaryote Kit v2 (Thermo Fisher Scientific, A15020) and processed using a previously published library preparation protocol[72]. Primers and adaptors used are listed in Table S1. The cDNA libraries were amplified using primers that carry Illumina indices, pooled, and 250-500 bp DNA fragments were isolated by agarose gel purification. The libraries were subjected to single-end 50-bp sequencing using the Illumina HiSeq 2000 platform. We utilized 2 to 3 biological replicates for each condition, each replicate contained tissue from 3 mice pooled together.

Supplementary Table 1

	Oligo	Sequence
Library Preparation	Reverse_Transcription_Hexamer	/5Phos/NNNNNN
	Reverse_Transcription_Octamer	/5Phos/NNWNNWNN
	Reverse_Transcription_PolyA	/5Phos/TTTTTTTTTVN
	Adaptor_Duplex_A_to_cDNA	5Phos/AGATCGGAAGAGCGTCGTGTAGG
	Adaptor_Duplex_B_to_cDNA_5N	CCCTACACGACGCUCTUCCGATCTNNNNN/3C6/
	Adaptor_Duplex_B_to_cDNA_6N	CCCTACACGACGCUCTUCCGATCTNNNNNN/3C6/
	Adaptor_Duplex_C_to_cDNA	GGAGTTCAGACGTGTGCTCTTCCGATCCTG
	Adaptor_Duplex_D_to_cDNA_5N	NNNNNCAGGAUCGGAAGAGCACACGUCTGAACTCC/3C6/
	Adaptor_Duplex_D_to_cDNA_6N	NNNNNNCAGGAUCGGAAGAGCACACGUCTGAACTCC/3C6/
	DLAR_Universal_Forward_Adaptor	AATGATACGGCGACCACCGAGATCTACACTCTTTCCCTACACGACGCTCTTCCGATC*T
	DLAF_Reverse_1	CAAGCAGAAGACGGCATACGAGATTGGTCAGTGACTGGAGTTCAGACGTGTGCTCTTCC
	DLAF_Reverse_2	CAAGCAGAAGACGGCATACGAGATCACTGTGTGACTGGAGTTCAGACGTGTGCTCTTCC
	DLAF_Reverse_3	CAAGCAGAAGACGGCATACGAGATATTGGCGTGACTGGAGTTCAGACGTGTGCTCTTCC
	DLAF_Reverse_4	CAAGCAGAAGACGGCATACGAGATGATCTGGTGACTGGAGTTCAGACGTGTGCTCTTCC
	DLAF_Reverse_5	CAAGCAGAAGACGGCATACGAGATTCAAGTGTGACTGGAGTTCAGACGTGTGCTCTTCC
	DLAF_Reverse_6	CAAGCAGAAGACGGCATACGAGATCTGATCGTGACTGGAGTTCAGACGTGTGCTCTTCC
	Sequencing_Primer	ACACTCTTTCCCTACACGACGCTCTTCCGATCT

Table S1. Primers and adaptors utilized in RNA-seq. All oligonucleotides are written in the 5' to 3’direction; *indicates a phosphorothioate bond; /5Phos/ indicates 5' phosphorylation; /3C6/ indicates a 3' hexanediol; red highlight indicates the reverse complement of the 6 bp-long Illumina sequencing index.

Supplementary Table 2

Dorsal hippocampus					
**Gene**	**Fold change**	**% of change**	**p-value**	**adjusted p**	**rt-qPCR**
Ano1	1.55	55.24	0.00	0.00	p>0.05
Col6a3	1.55	55.16	0.00	0.00	p>0.05
Grm4	1.51	51.38	0.00	0.00	p>0.05
Calb2	1.51	50.74	0.00	0.00	p>0.05
Ebf2	1.50	50.24	0.00	0.00	
Trpc3	1.50	49.96	0.00	0.00	
Scn5a	1.45	45.34	0.00	0.00	
Slc8a3	1.45	44.96	0.00	0.00	*p<0.05
Ebf3	1.45	44.69	0.00	0.00	
Man1a	1.43	43.45	0.00	0.00	
Rcan2	1.43	43.00	0.00	0.00	
Npas2	1.43	42.70	0.00	0.01	
Chrm2	1.41	41.13	0.00	0.02	p>0.05
Tmsb10	1.41	41.10	0.00	0.02	
Zic2	1.41	40.81	0.00	0.02	
Cdh6	1.40	40.31	0.00	0.03	
Plekha7	1.40	40.03	0.00	0.03	
Pld5	1.39	38.80	0.00	0.03	
Ank1	1.38	38.33	0.00	0.02	
Rmst	1.37	37.13	0.00	0.01	
Tmem56	1.37	37.09	0.00	0.01	
Adcy8	1.37	36.97	0.00	0.02	**p<0.01
Astn2	1.36	35.99	0.00	0.00	
Zic4	1.35	35.37	0.00	0.02	
Cpa6	1.35	35.25	0.00	0.05	
Sez6	1.35	34.63	0.00	0.05	
Pex5l	1.32	31.55	0.00	0.01	
Hunk	1.31	31.30	0.00	0.02	
Gpr88	1.31	31.15	0.00	0.02	
Met	1.29	29.23	0.00	0.05	
Arid5b	1.28	27.95	0.00	0.04	
Glp1r	1.27	26.80	0.00	0.01	p>0.05
Kcnip1	1.26	26.10	0.00	0.03	
Farp1	1.25	25.03	0.00	0.03	
Slc17a7	0.80	-19.69	0.00	0.05	
Zcchc16	0.78	-21.60	0.00	0.04	
Cpne6	0.77	-23.29	0.00	0.02	
Pcdh20	0.76	-23.51	0.00	0.02	
Tspan18	0.75	-25.09	0.00	0.04	
Nptx1	0.75	-25.26	0.00	0.02	
Ak4	0.75	-25.37	0.00	0.05	
Neto1	0.75	-25.37	0.00	0.01	**p<0.01
Npy2r	0.71	-28.53	0.00	0.02	p>0.05
Homer3	0.71	-28.61	0.00	0.02	
Arc	1.25	25.46	0.00	0.39	**p<0.01
Egr1	1.21	20.71	0.01	0.74	*p<0.05

Medial prefrontal cortex					
**Gene**	**Fold change**	**% of change**	**p-value**	**adjusted p**	**rt-qPCR**
Fkbp5	1.37	36.65	0.00	0.00	*p<0.05
Slc2a1	1.30	29.72	0.00	0.01	p>0.05
Htra1	1.27	26.88	0.00	0.04	
Chl1	0.85	-15.04	0.00	0.04	
Ldb2	0.85	-15.18	0.00	0.05	
Tenm2	0.84	-16.18	0.00	0.03	
Tox	0.83	-16.53	0.00	0.04	
Egfem1	0.82	-17.56	0.00	0.00	
Zfp804b	0.75	-24.57	0.00	0.00	
Arc	1.14	14.47	0.02	0.37	*p<0.05
Egr1	1.14	14.26	0.02	0.39	*p<0.05

Table S2. List of differentially expressed genes in the DH and mPFC following alcohol memory retrieval. The table contains estimates for the fold change and percent of change in the expression of differentially expressed genes in the Retrieval group, as compared to No Retrieval group. The table also lists differentially expressed genes validated by qRT-PCR analysis.

Supplementary Figure 1

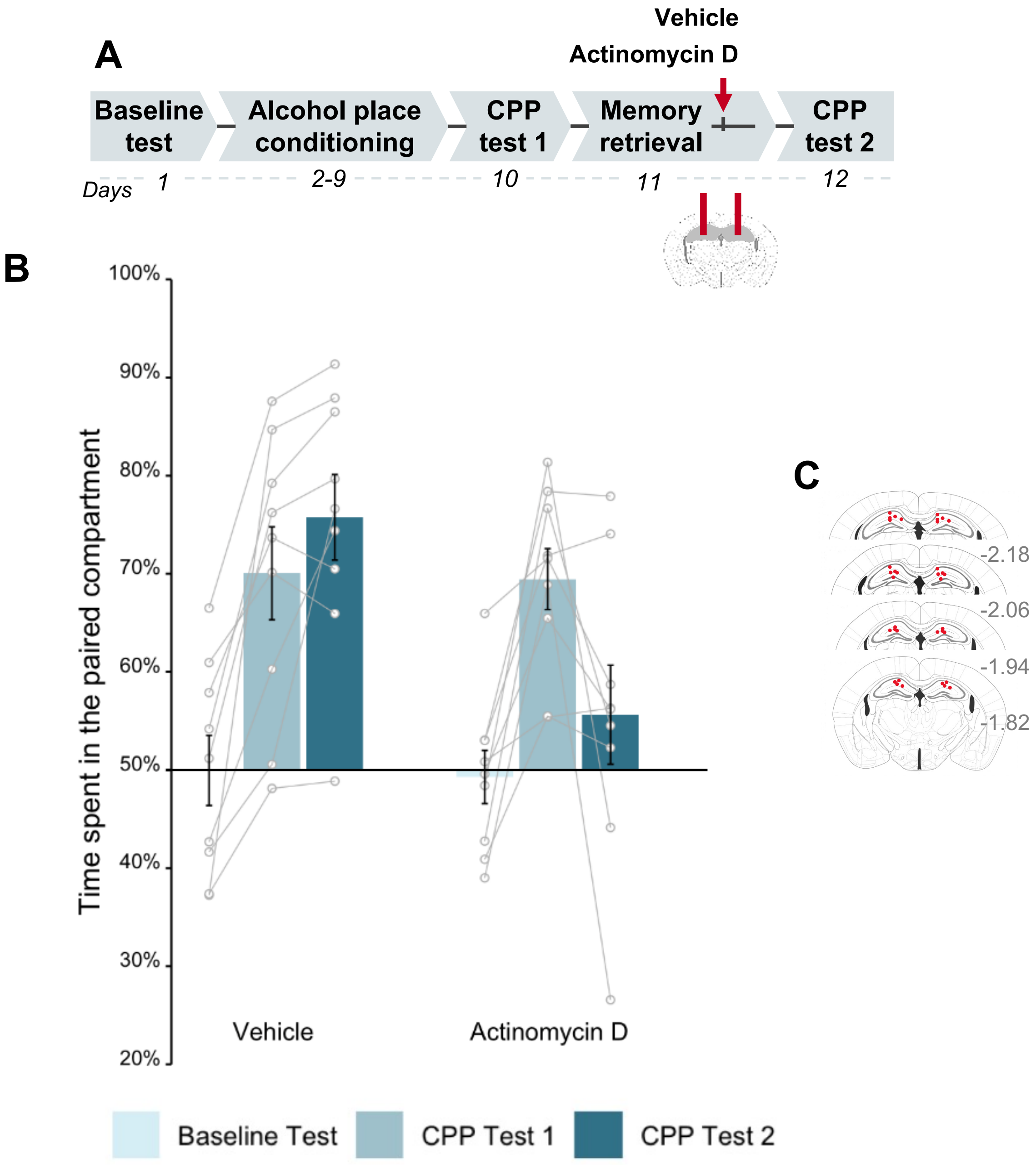

Figure S1. Inhibition of transcription in the dorsal hippocampus after alcohol memory retrieval disrupts the expression of alcohol-CPP. A. Schematic illustration of the experimental design and timeline. Actinomycin D (4 µg/µl) was bilaterally infused into the dorsal hippocampus of mice immediately following the retrieval of alcohol memories. **B.** Place preference scores, expressed as means ±S.E.M. of the percent of time spent in the alcohol-paired compartment. **C**. Locations of cannulas. *$p<0.05$; n=9 per group.

Supplementary Figure 2

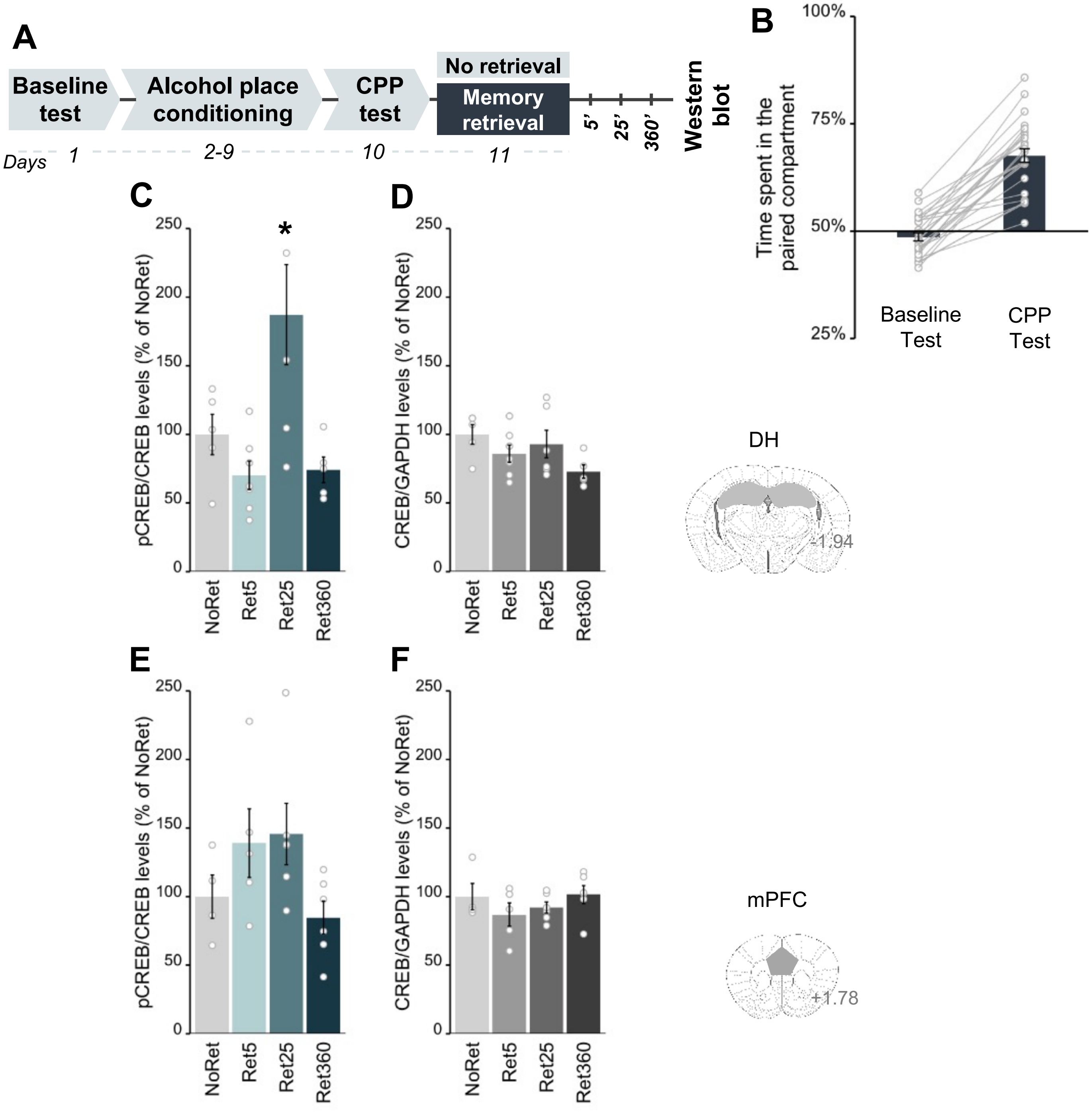

Figure S2. Alcohol memory retrieval induces CREB phosphorylation in the DH and mPFC. A. Schematic illustration of the experimental design and timeline. **B.** Place preference scores, expressed as means ±S.E.M.. of the percent of time spent in the alcohol-paired compartment; **C-F.** Protein levels, expressed as means ±S.E.M. of the percent of change from the control group (No Retrieval). Levels of pCREB were normalized to of tCREB in the DH. The levels of pCREB in the DH were increased 25 min after alcohol-memory retrieval (one-way ANOVA; Time ($F_{(3,18)}$=6.07, $p<0.05$); post hoc: NoRet vs Ret25' ($p<0.05$)) (**C**) or mPFC (**E**). Levels of tCREB were normalized to GAPDH in the DH (**D**) or mPFC (**F**).; *$p<0.05$; n=5-6 per group.

Supplementary Figure 3

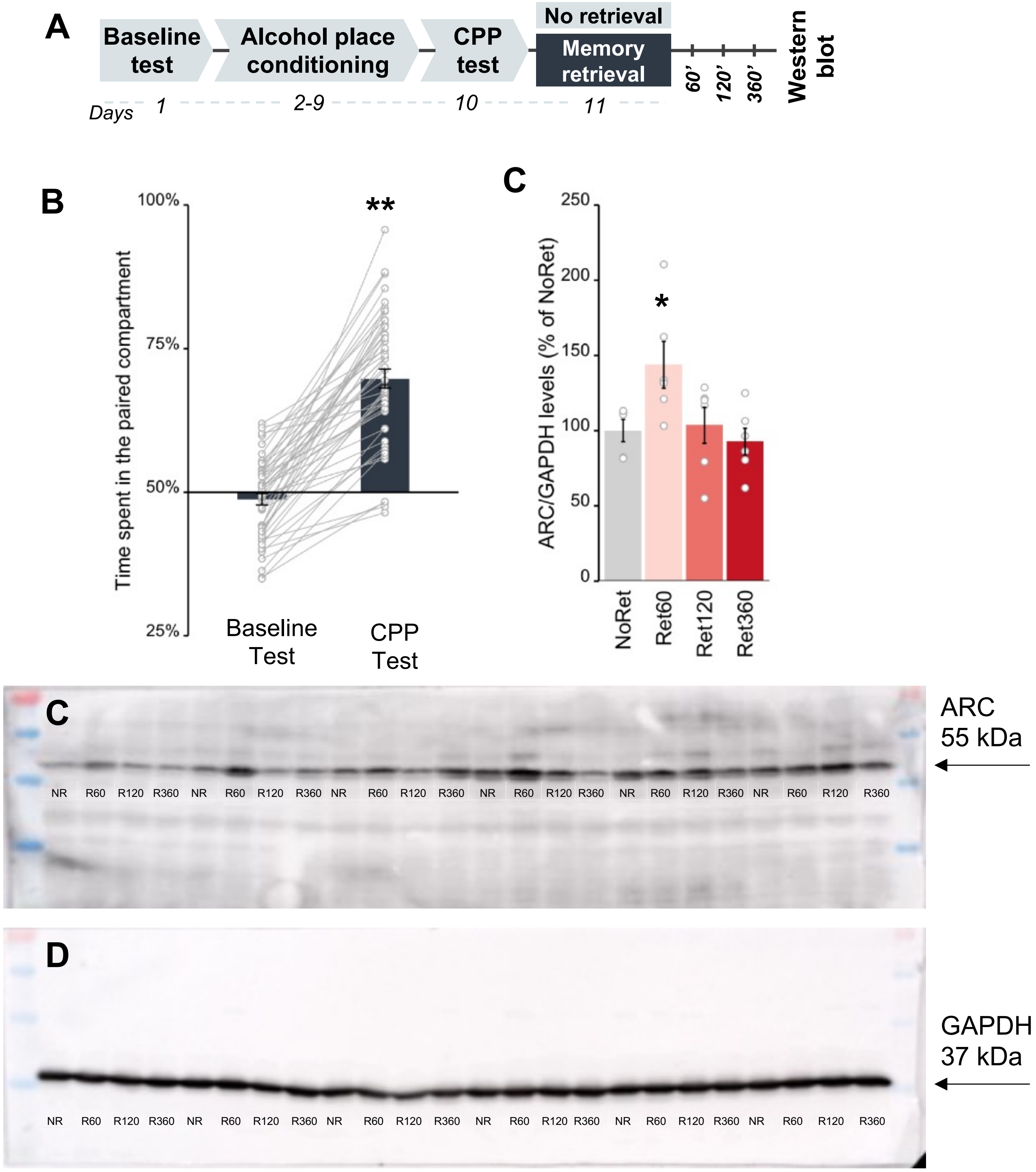

Figure S3. Alcohol memory retrieval induces upregulation of ARC protein expression in the DH. A. Schematic illustration of the experimental design and timeline. **B.** Place preference scores, expressed as means ±S.E.M. of the percent of time spent in the alcohol-paired compartment ($t(25)=9.41$, $p<0.0001$); C. ARC protein levels, normalized to GAPDH, expressed as means ±S.E.M. of the percent of change from the control group (No Retrieval). The levels of ARC protein in the DH were increased 60 min after alcohol memory retrieval (one-way ANOVA; Time ($F(3,19)=3.94$, $p<0.05$); post hoc: NoRet vs Ret60' ($p<0.05$)). ARC (**C**) and GAPDH (**D**) protein expression in the DH following alcohol-memory retrieval, as revealed by Western blot. *$p<0.05$; **$p<0.01$; n=6-7 per group.

Supplementary Figure 4

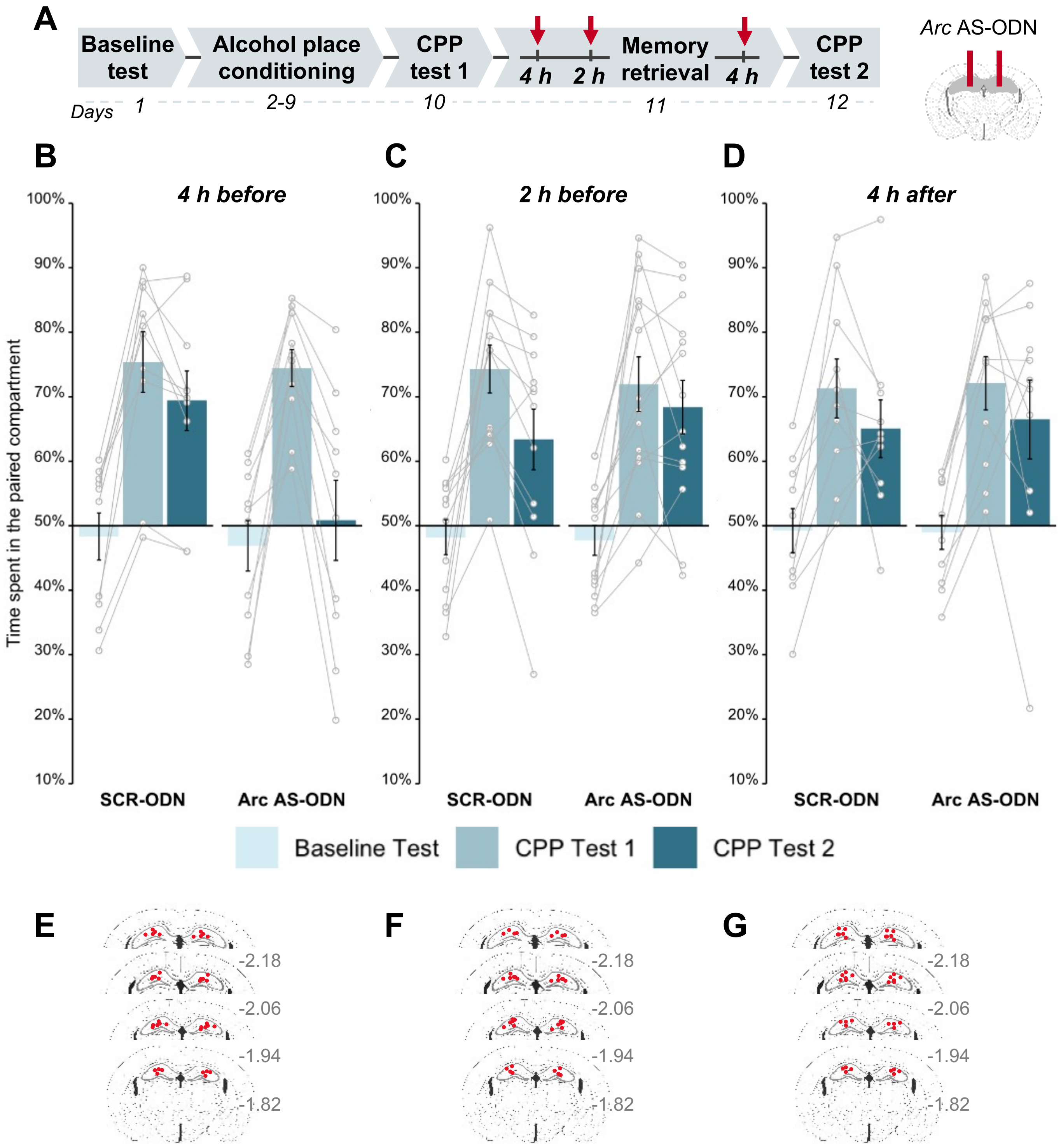

Figure S4. Downregulation of ARC protein in the dorsal hippocampus shortly after alcohol memory retrieval disrupts the expression of alcohol-CPP. A. Schematic illustration of the experimental design and timeline. Antisense oligodeoxynucleotides directed against *Arc* mRNA (*Arc* AS-ODN) or non-specific scrambled oligodeoxynucleotides (SCR-ODN) were infused into the dorsal hippocampus (DH) of mice at the indicated time points. **B-D.** Place preference scores, expressed as means ±S.E.M. of the percent of time spent in the alcohol-paired compartment. Infusion of *Arc* AS-ODN disrupted the expression of alcohol-CPP only when infused 4 h **(B),** but not 2 h before memory retrieval **(C)** or 4 h after memory retrieval **(D)**, as compared with SCR-ODN-treated controls. **E-G.** Locations of cannulas. *$p<0.05$; n=10-12 per group.

Supplementary Figure 5

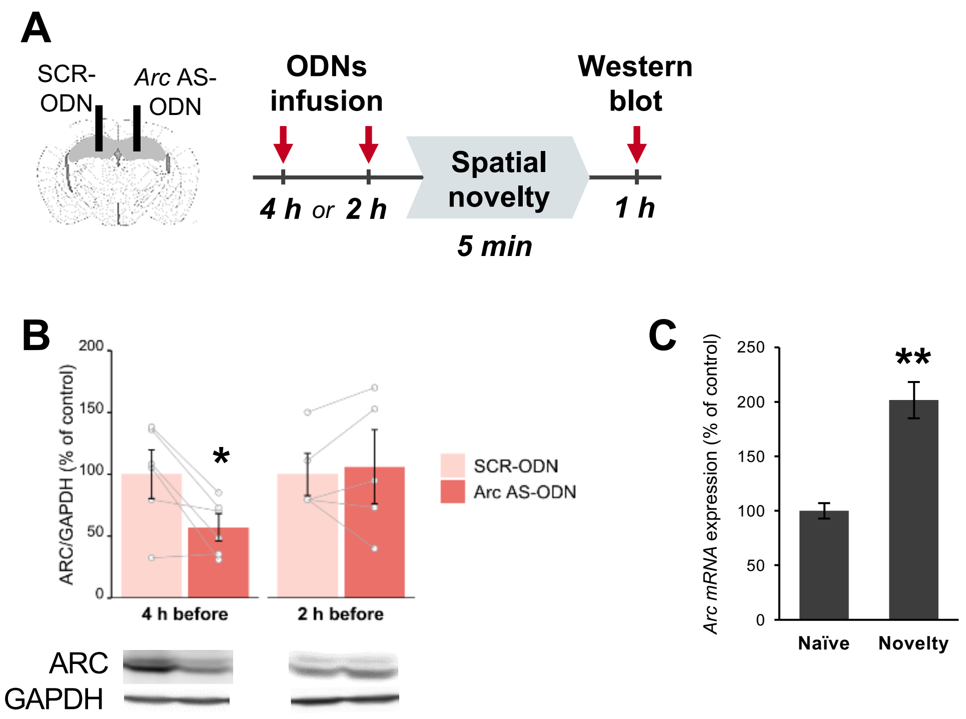

Figure S5. Validation of *Arc* AS-ODN. A. Schematic representation of the validation of the ability of *Arc* AS-ODN to downregulate ARC protein expression in the DH. To this end, we infused *Arc* AS-ODN (2 nmol/µl, 0.5 µl) into one hemisphere, and an equivalent amount of a non-specific SCR-ODN into the other hemisphere (sides were counter-balanced). Two or four hours later, we allowed the mice to explore an unfamiliar environment (CPP compartment) for 5 min to induce novelty-dependent ARC expression. One hour later (3 or 5 h after ODN infusion), brain tissues were collected, and the levels of ARC protein were assessed by Western blot. **B.** Protein levels, expressed as means ±S.E.M. of the percent of change from the control hemisphere (SCR-ODN). Levels of ARC were normalized to GAPDH. We found that *Arc* AS-ODN produced a 40% decrease in novelty-induced ARC protein levels in the DH, when administered 4 h ($t_{(5)}=-3.29$, $p<0.05$), but not 2 h ($t_{(5)}=0.44$, $p>0.05$), prior to the novelty presentation, as compared to the SCR-ODN control. These results show that infusion of *Arc* AS-ODN into the DH downregulated ARC protein levels 5 h, but not 3 h, after infusion. **C.** mRNA levels, normalized to *Gapdh*, as the percent of change from the control group (naive). qRT-PCR analysis revealed a rapid 2-fold change in *Arc* expression in the DH in mice 30 min after a 5 min novelty exploration period ($p<0.01$). *p<0.05; **p<0.01; n=4-6 per group.

Supplementary Figure 6

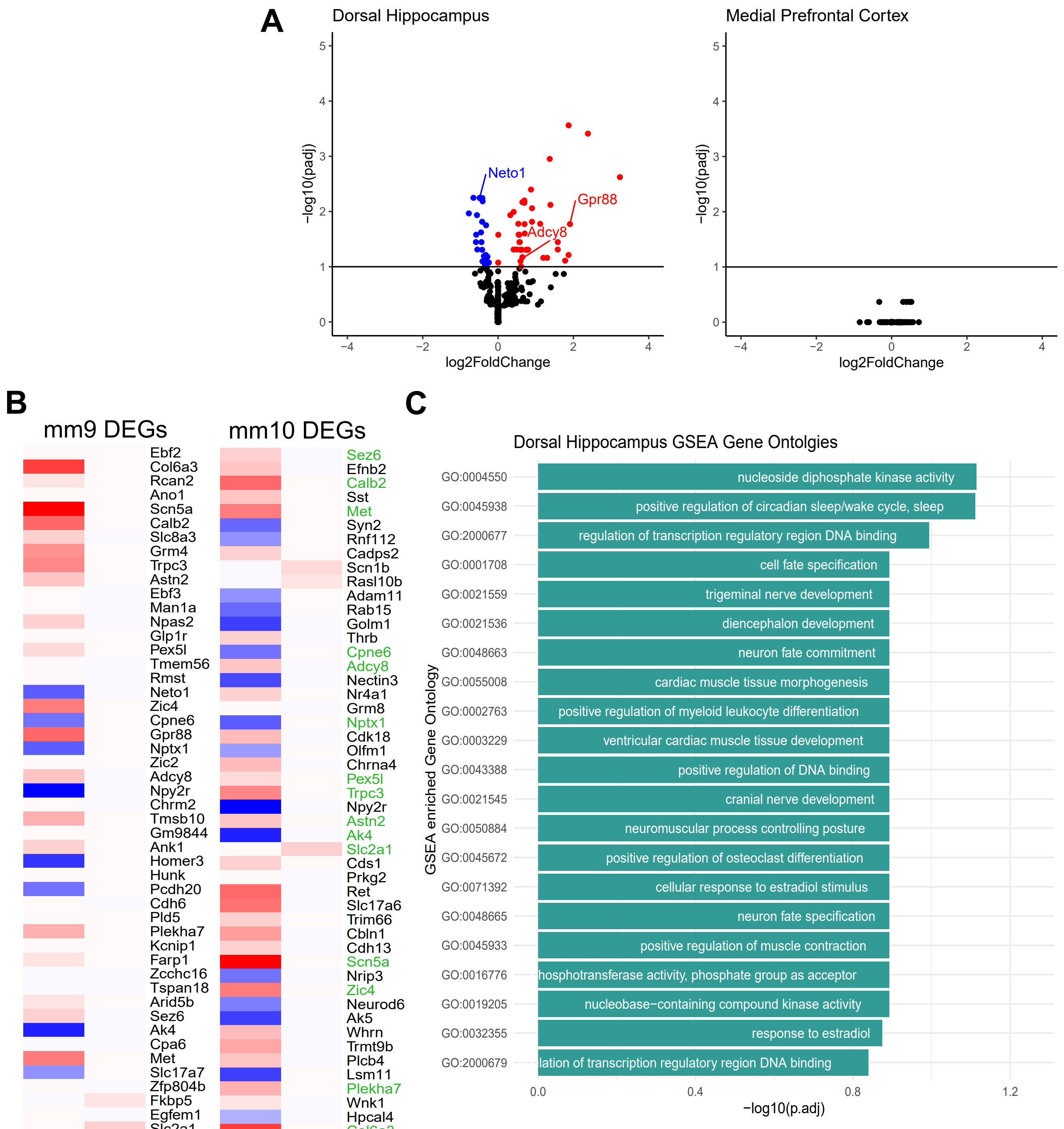

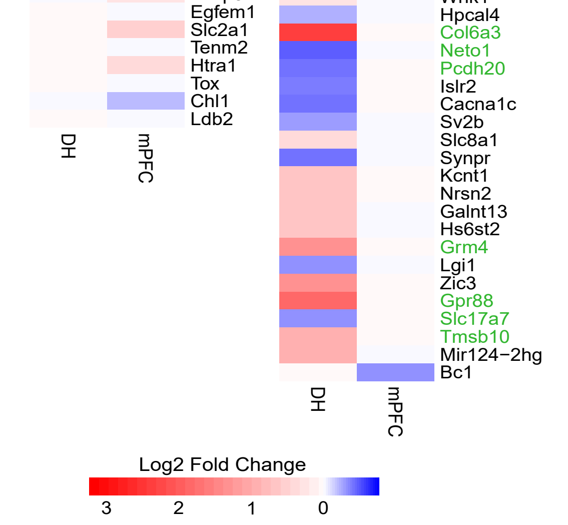

Figure S6. Gene expression changes aligned to mm10 in the dorsal hippocampus and medial prefrontal cortex during alcohol-memory retrieval. (A) Volcano plots of significantly upregulated (red) and downregulated (blue) genes (q<0.1). (B) Heatmaps of differentially expressed genes (DEGs) called in the mm9 (left) and mm10 (right) alignment. Color is log2fold change based on mm10 alignment and DESeq2 analysis. DEGs common between the mm10 and mm9 alignments are highlighted in green. (C) Top gene ontologies called from gene set enrichment analysis (GSEA, clusterProfiler) of global gene expression changes in the dorsal hippocampus.
